# Supplementary material for: CD34+CD38−CD123+ Leukemic Stem Cell Frequency Predicts Outcome in Older Acute Myeloid Leukemia Patients Treated by Intensive Chemotherapy but Not Hypomethylating Agents
Source: Cancers (Basel). 2020 May 6;12(5):1174. doi: 10.3390/cancers12051174 (PMC7281486; doi:10.3390/cancers12051174)
Supplement: Supplementary file 1 [file cancers-12-01174-s001.zip › Supplementary Figure 1.pptx]

## Slide 1
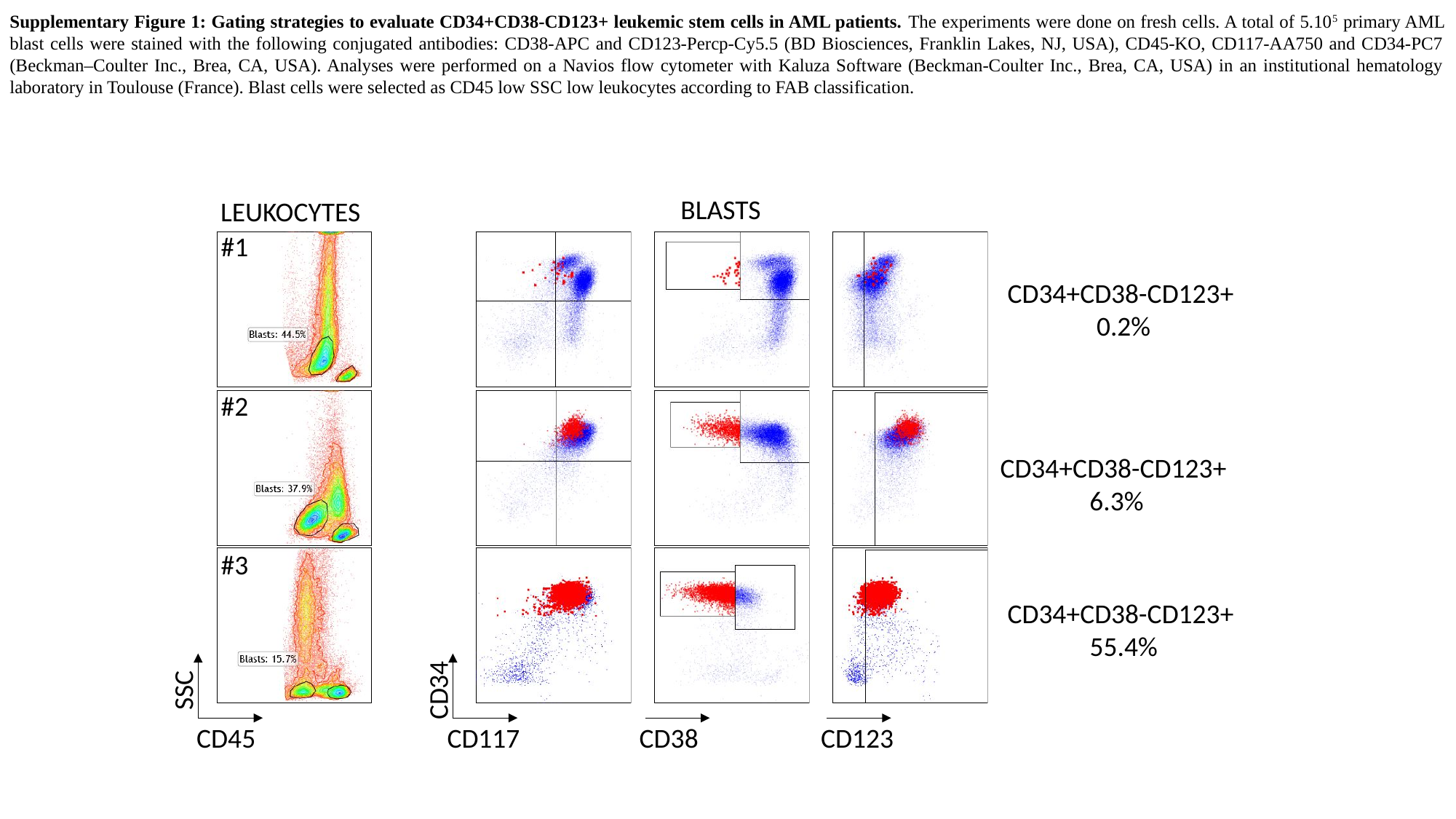

Supplementary Figure 1: Gating strategies to evaluate CD34+CD38-CD123+ leukemic stem cells in AML patients. The experiments were done on fresh cells. A total of 5.105 primary AML blast cells were stained with the following conjugated antibodies: CD38-APC and CD123-Percp-Cy5.5 (BD Biosciences, Franklin Lakes, NJ, USA), CD45-KO, CD117-AA750 and CD34-PC7 (Beckman–Coulter Inc., Brea, CA, USA). Analyses were performed on a Navios flow cytometer with Kaluza Software (Beckman-Coulter Inc., Brea, CA, USA) in an institutional hematology laboratory in Toulouse (France). Blast cells were selected as CD45 low SSC low leukocytes according to FAB classification.
BLASTS
LEUKOCYTES
#1
CD34+CD38-CD123+
0.2%
#2
CD34+CD38-CD123+
6.3%
#3
CD34+CD38-CD123+
55.4%
CD34
CD117
SSC
CD45
CD38
CD123
