# Supplementary material for: CD34+CD38−CD123+ Leukemic Stem Cell Frequency Predicts Outcome in Older Acute Myeloid Leukemia Patients Treated by Intensive Chemotherapy but Not Hypomethylating Agents
Source: Cancers (Basel). 2020 May 6;12(5):1174. doi: 10.3390/cancers12051174 (PMC7281486; doi:10.3390/cancers12051174)
Supplement: Supplementary file 1 [file cancers-12-01174-s001.zip › Supplementary Figure 2.pptx]

## Slide 1
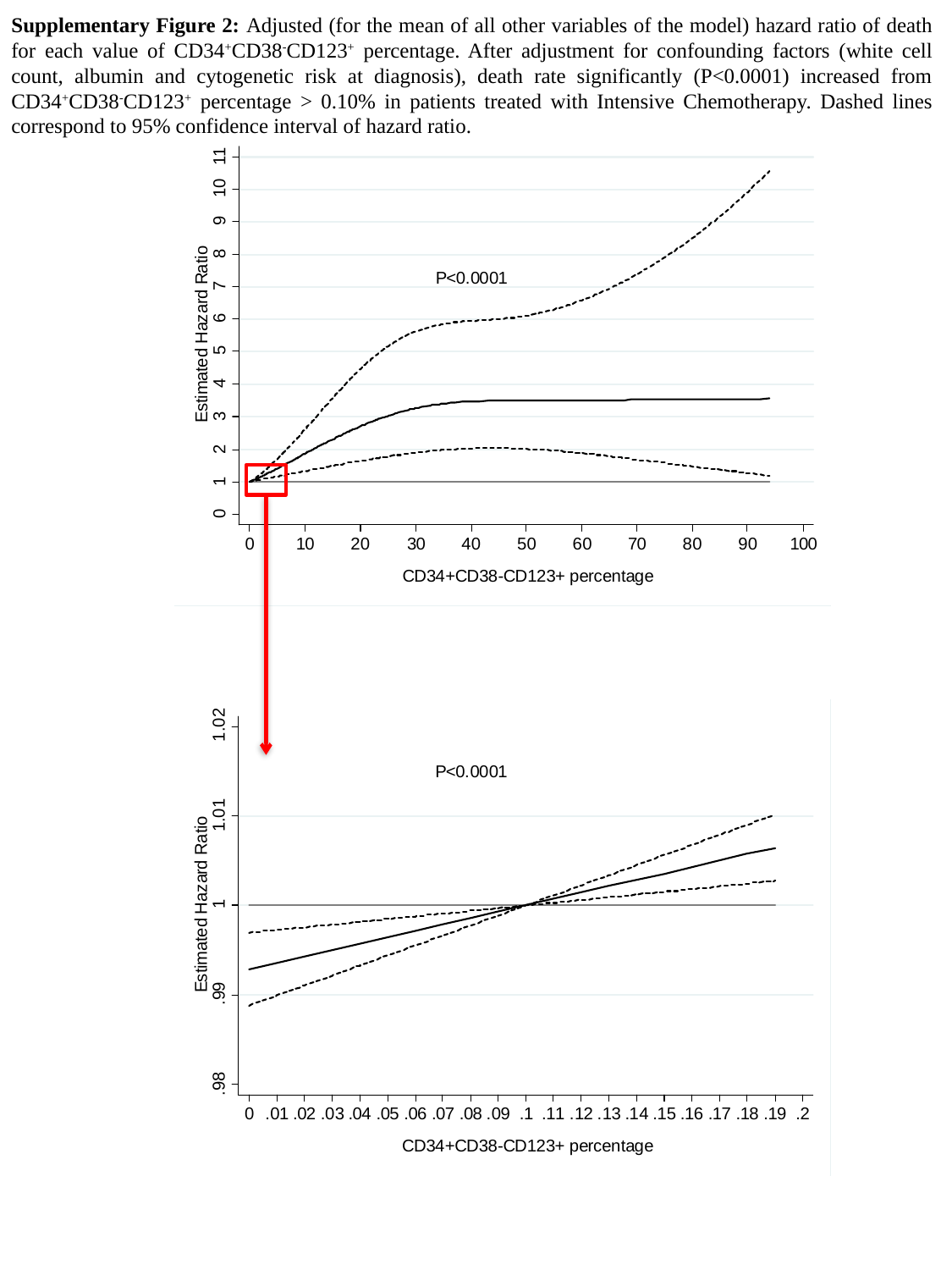

Supplementary Figure 2: Adjusted (for the mean of all other variables of the model) hazard ratio of death for each value of CD34+CD38-CD123+ percentage. After adjustment for confounding factors (white cell count, albumin and cytogenetic risk at diagnosis), death rate significantly (P<0.0001) increased from CD34+CD38-CD123+ percentage > 0.10% in patients treated with Intensive Chemotherapy. Dashed lines correspond to 95% confidence interval of hazard ratio.
