# Supplementary material for: CD34+CD38−CD123+ Leukemic Stem Cell Frequency Predicts Outcome in Older Acute Myeloid Leukemia Patients Treated by Intensive Chemotherapy but Not Hypomethylating Agents
Source: Cancers (Basel). 2020 May 6;12(5):1174. doi: 10.3390/cancers12051174 (PMC7281486; doi:10.3390/cancers12051174)
Supplement: Supplementary file 1 [file cancers-12-01174-s001.zip › Supplementary Figure 4.pptx]

## Slide 1
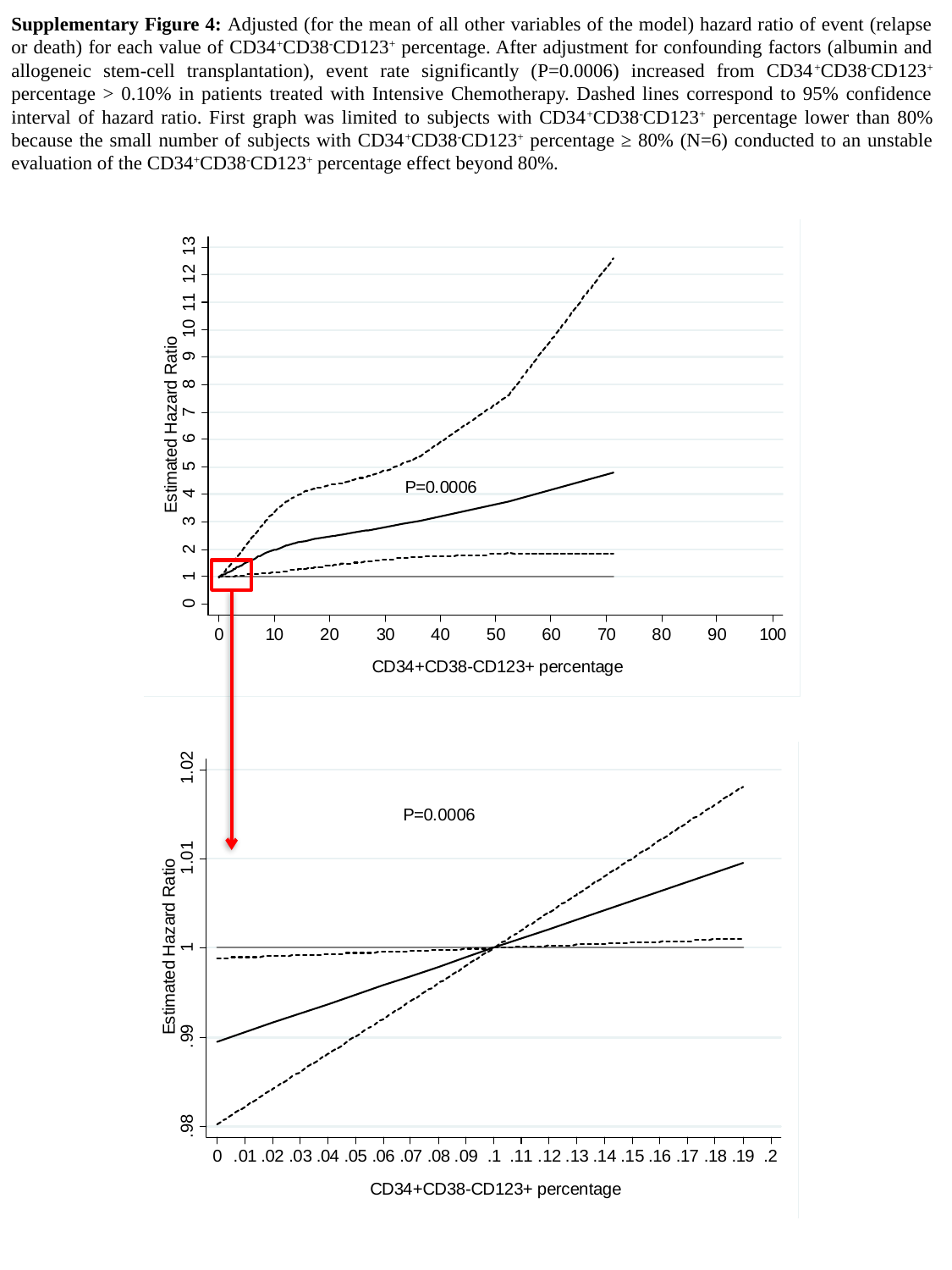

Supplementary Figure 4: Adjusted (for the mean of all other variables of the model) hazard ratio of event (relapse or death) for each value of CD34+CD38-CD123+ percentage. After adjustment for confounding factors (albumin and allogeneic stem-cell transplantation), event rate significantly (P=0.0006) increased from CD34+CD38-CD123+ percentage > 0.10% in patients treated with Intensive Chemotherapy. Dashed lines correspond to 95% confidence interval of hazard ratio. First graph was limited to subjects with CD34+CD38-CD123+ percentage lower than 80% because the small number of subjects with CD34+CD38-CD123+ percentage ≥ 80% (N=6) conducted to an unstable evaluation of the CD34+CD38-CD123+ percentage effect beyond 80%.
